# Surveillance of surgical site infections: methodical comparison of the IQTIG and KISS strategies

**DOI:** 10.3205/dgkh000389

**Published:** 2021-05-04

**Authors:** Sascha Rittmeier, Reiner M. Waeschle, Tanja Artelt, Patrick Fehling, Arnt Suckow, Martin Siess, Simone Scheithauer

**Affiliations:** 1 Institute for Infection Control and Infectious Diseases, University Medical Center Goettingen, Goettingen, Germany; 2 Department of Anesthesiology, University Medical Center Goettingen, Goettingen, Germany; 3 Quality and Risk Management Department, University Medical Center Goettingen, Goettingen, Germany; 4 Board of Health Care, University Medical Center Goettingen, Goettingen, Germany

**Keywords:** surgical wound, surgical wound infection, infection surveillance, IQTIG, KISS

## Abstract

**Aim:** In 2017, the Institute for Quality Assurance and Transparency in Healthcare (IQTIG) introduced a quality assurance system for the surveillance of surgical site infections (SSI) on behalf the Federal Joint Committee. The establishment of the new system was made in parallel to existing methods, such as the “Krankenhaus-Infektions-Surveillance-System” (KISS). The aim of this work was to perform a comparative analysis.

**Methods:** All 2,233 cases at the University Medical Center Goettingen requiring an assessment of the presence of SSI as part of the IQTIG procedure in 2018 and 2019 were evaluated retrospectively according to the KISS protocol.

**Results:** In total, 2,050 patients were included in the comparative evaluation. Overall, 1,779 (79.7%) had a surgical anamnesis (surgery during the stay or in the past), and 1,716 (83.7%) showed identical results for both surveillance strategies. Different results were found for 334 patients (16.3%), with 160 of these (7.8%) positive for SSI according to IQTIG and 174 (8.5%) positive for KISS. Risk factors were identified for a discordant assessment between the methods.

**Conclusion:** The congruence of the two strategies was consistently high over the study period. There is evidence that the efficiency of the documentation algorithm can be increased without the loss of documentation of SSI, while preserving the precision of the documentation through training.

## Zusammenfassung

**Zielsetzung:** Zur Surveillance postoperativer Wundinfektionen wurde 2017 im Auftrag des Gemeinsamen Bundesausschusses ein sektorenübergreifendes Qualitätssicherungsverfahren durch das Institut für Qualitätssicherung und Transparenz im Gesundheitswesen (IQTIG) eingeführt (*Vermeidung nosokomialer Infektionen – postoperative Wundinfektionen, QS WI*). Die Etablierung erfolgte parallel zu bestehenden Methoden wie z.B. des Krankenhaus-Infektions-Surveillance-Systems (KISS). Das Ziel dieser Arbeit war die vergleichende Analyse dieser beiden Methoden.

**Methodik:** Alle 2.233 Patientenfälle, die im Rahmen des IQTIG QS-Verfahrens 2018 und 2019 an der Universitätsmedizin Göttingen (UMG) eine Bewertung bzgl. Vorhandenseins postoperativer Wundinfektionen erforderten, wurden retrospektiv gemäß den Vorgaben des KISS-Protokolls nachbeurteilt und abschließend mit den Ergebnissen der IQTIG-Methodik verglichen.

**Ergebnisse:** 2.050 Fälle konnten in die vergleichende Auswertung einbezogen werden. 1.779 (79,7%) wiesen einen primären chirurgischen Bezug auf (OP während des Aufenthalts/zuvor/ggf. extern). 1.716 (83,7%) zeigten identische Resultate bei den Bewertungen durch die beiden Surveillance-Systeme. Bei 334 (16,3%) kam es zu abweichenden Ergebnissen, wobei sich 160 (7,8%) mit einer positiven Wundinfektionsbeurteilung nach IQTIG und 174 (8,5%) mit einer positiven Bewertung nach KISS ergaben. Es fanden sich verschiedene Risikofaktoren für eine unterschiedliche Beurteilung zwischen den Verfahren.

**Fazit:** Die Kongruenz beider Strategien zeigt sich über den Beobachtungszeitraum konstant. Es konnten Hinweise zur Optimierung der Effizienz des potenziell zu breit angelegten Auslösealgorithmus im QS WI-Verfahren ohne Verlust der Dokumentation postoperativer Wundinfektionen identifiziert werden Zudem zeigt sich Potenzial zur Steigerung der Dokumentationspräzision durch Schulungsmaßnahmen.

## Introduction

Surgical site infections are one of the most common complications of postsurgical patient care among nosocomial infections. As one of the few parameters of medical quality assurance, their occurrence can be assessed by both healthcare professionals and patients. Current point prevalence studies with data from 29 European countries put the estimated prevalence of nosocomial infections in acute care hospitals at 6.5% [1]. The prevalence of SSI in Germany is approximately 4.6% [2]. In addition, surgical site infections result in an estimated average prolongation of the inpatient stay of 4 days [3] as well as an additional financial burden of up to €30,000 per individual case [4]. Other international research identified annual costs of hospital-acquired infections as 9.8 billion dollars, “with surgical site infections contributing the most to overall costs (33.7% of the total)” [5]. The costs on a per-case basis for surgical site infections was calculated in that study to be around $20,785 [5].

Many studies have now provided evidence that sufficient surveillance is a suitable tool to significantly reduce all nosocomial infection rates [6], [7]. 

### IQTIG and KISS

At the beginning of 2017, the Institute for Quality Assurance and Transparency in Health Care (IQTIG) implemented the quality assurance procedure for “Prevention of nosocomial infections – surgical site infections” (QA-SSI). With the components of case- and institution-related QA documentation as well as subsequent linking to relevant social data, the procedure is intended to generate cross-sectoral surveillance of corresponding surgical patient cases in the outpatient and inpatient sectors https://iqtig.org/downloads/berichte/2016/IQTIG_Poster_ QS-WI_2017-09-28.pdf [8], [9]. Based on the service and routine data in accordance with § 21 KHEntgG (German Hospital Fee Act), algorithms and combinations of different codes are used to trigger a documentation obligation to assess potential surgical site infections [8], [9]. The staff of the medical department to which the patient belongs at the time of initiation is responsible for meeting this requirement [8]. This strategy is to be used prospectively for overarching control.

In contrast, the well-proven method corresponds to the surgical module of the German Hospital Infection Surveillance System (KISS). The surveillance system, which is based on the principles and definitions of the American Centers for Disease Control and Prevention (CDC), includes a collection of surgery-relevant data on corresponding indicator operations by specially trained hygiene personnel [10], [11]; the population is a group of similar patients selected by a specific surgery according to a given surgical procedure. This strategy is usually used for in-house and departmental optimization.

The primary aim of this research project was to compare the two methods in the evaluation of patient cases with potential surgical site infections within the population of cases initiated via IQTIG.

The objectives are also directly related to similar existing research approaches at the international level [12]. That study, which was very similar in nature to the current work, showed significant discrepancies between the procedures, which were also partly based on the use of administrative data [12]. In this respect, it will be interesting to analyze to what extent these results are also confirmed or different in the intended comparison between IQTIG and the German national KISS system.

## Methods

For the methodological comparison, the total number of cases with documentation obligation on the part of the IQTIG QA-SSI procedure between 2018 and 2019 at the University Medical Center of Goettingen (UMG) was used (n=2,233). The UMG performs more than 25,000 surgical procedures on approximately 22,000 patient cases annually (status as of 08/2020). To ensure data protection, the project was coordinated with the head of data protection officer prior to implementation. The implementation was supported by the entire board of directors; all clinic directors as well as the nursing service management were informed about the project in advance. 

The population of cases in question was provided by the staff unit of the Quality and Risk Management Department of the UMG. All selected cases were retrospectively evaluated by a physician trained in standardized surveillance analogous to the surgical module of KISS. Unclear cases were discussed with at least two infection-control specialists with many years of surveillance experience and evaluated in consensus. The results of the evaluations of the two procedures regarding potential surgical site infections were compared, and concordance and deviation were documented. 

The group assessed as concordant was compared with the group assessed as discordant and examined for distinguishing features. Subsequently, within the group assessed as discordant, the subgroup of those positively evaluated according to IQTIG and the subgroup of those positively evaluated according to KISS were compared and examined for distinguishing characteristics according to the previous procedure. 

The data relevant for the KISS evaluation were exported based on patient case lists from the hospital information systems SAP IS-H (EHP 6 for ERP 6.0; Siemens), from ixserv (4.25; ix.mid Software Technologie GmbH) and HyBASE-Administrator-Klinik (V6.2020.03.R09; epiNET AG) and summarized in Excel 2016 (16.0; Microsoft).

In addition to the standard data collection, data items were determined in order to explain possible differences and classify them more precisely.

Consecutive statistical analysis was performed in cooperation with the Institute of Medical Statistics and the Clinical Trial Center and with SPSS software (Version 26.0; IBM Corp. released 2019, Armonk, NY) to determine the odds ratio (OR), associated 95% confidence interval [95%-CI] and p-value according to the Fisher’s exact test (significance level at p<0.05). When indicated, a Bonferroni correction for multiple testing was performed.

## Results

### General information

Overall, 1,273 (57%) patients were male and 960 (43%) were female. The mean age of all patients at the time of the respective inpatient stay was 63.8 years, with a standard deviation of s=16.8 years.

As a result of underdocumentation of the IQTIG procedure, not all cases could be used for the subsequent comparison of results, and the number of cases was reduced to 2,050, with a cumulative documentation rate of 91.4% (2018: 90.5%; 2019: 92.3%).

139 (6.2%) of all cases had no surgical reference, 454 (20.3%) had a previous (externally performed) surgery and 1,640 (73.4%) patient cases had surgery at UMG during their inpatient stay. Table 1 (Tab. 1) provides a detailed overview of all surgical cases.

### Comparison of the assessment results of the two surveillance strategies

**Figure 1:**
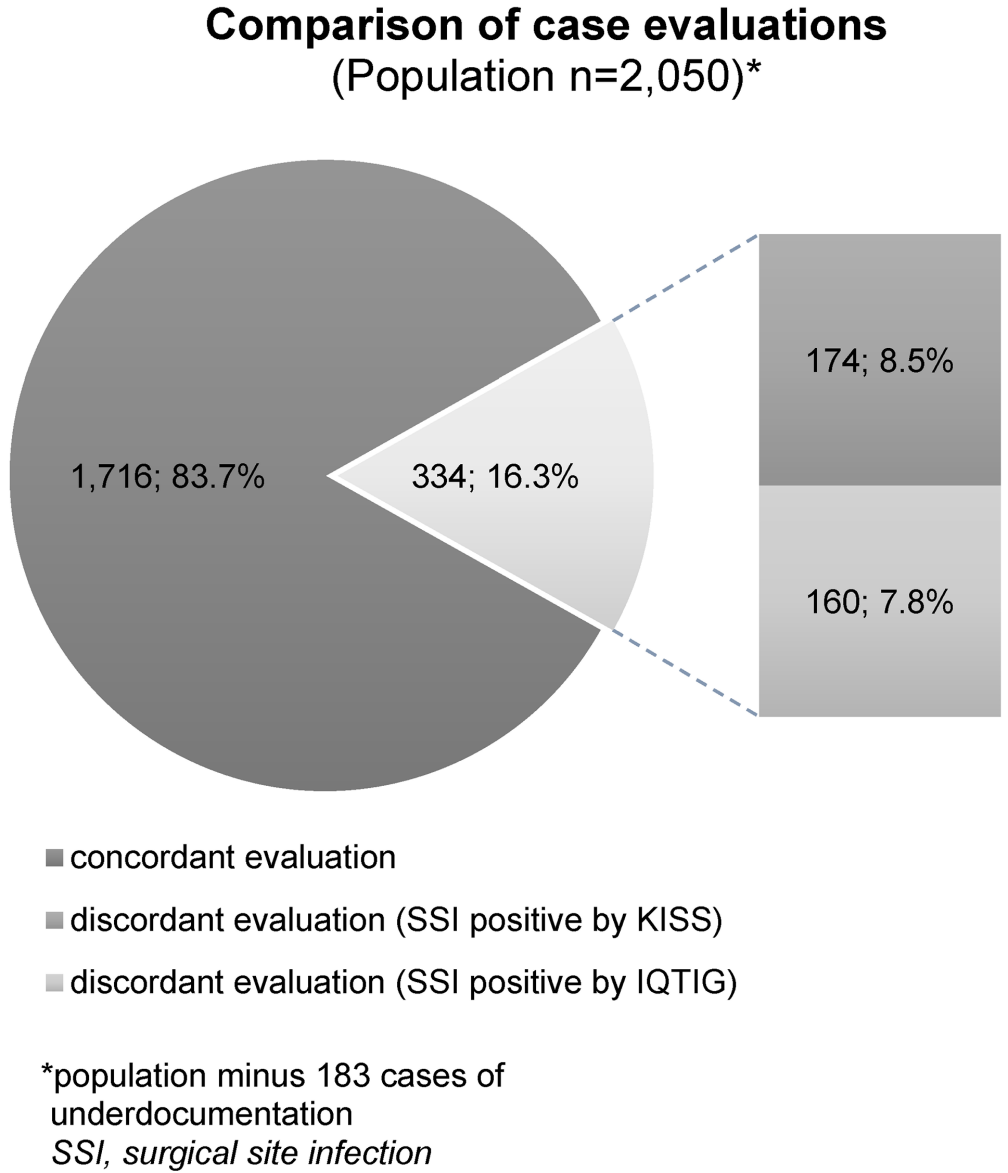
Comparison of case evaluations regarding the occurrence of surgical site infections by IQTIG and KISS

Comparison of the case evaluations by the IQTIG and KISS strategies is shown in Figure 1(Fig. 1). The congruence rate of the groups assessed as concordant was also constant when comparing the years 2018 and 2019 (2018: 941/1,124 (83.7%); 2019: 775/926 (83.7%)).

In a secondary evaluation step, potential risk factors or significant differences were identified between the groups assessed as discordant vs. groups assessed as concordant.

The examined risk factors and corresponding case numbers are shown in Table 2 (Tab. 2).

From the comparative data listed in table form, some of the examined risk factors with significant anomalies can be identified. 

The intergroup comparison showed significantly more cases with primary surgical references within the case group assessed as discordant. In addition, the total number of recorded surgical site infections, both on the basis of the IQTIG procedure and according to the KISS assessment, was higher in this case group than in the group of patients evaluated as concordant.

The surveillance time of cases within the group assessed as discordant was terminated significantly more often in the odds-ratio test due to resurgery in the primary surgical area. However, this difference was not significant after the Bonferroni correction was performed.

### Comparison within the case group assessed as discordant

In accordance with the procedure described above, a comparison was then carried out within the totality of the cases that were assessed as discordant (see Figure 1, n=334).

This intragroup examination of possible risk factors was performed between the cases that were positively evaluated regarding surgical site infections in the respective surveillance system (cf. Figure 1: SSI positive according to IQTIG, n=160 vs. SSI positive according to KISS, n=174).

The schedule in Table 3 (Tab. 3) presents all the factors determined within the analysis that showed conspicuities in the comparison.

In addition to the factors that occurred more frequently in the group of cases assessed as positive for surgical site infections according to IQTIG (resurgery in the primary surgical area as the reason for the termination of surveillance and pathogen detection in the blood culture), further abnormalities were found when considering coding-relevant data. 

Some of the listed special procedure classification codes (German OPS codes) and codes of the International Statistical Classification of Diseases and Related Health Problems (ICD-10-GM codes) were identified that coded significantly more often in cases in one subgroup or another.

### Examination of the population generated by the IQTIG QA procedure

The information in Table 4 (Tab. 4) shows that 20.3% of all patient cases with an obligation of documentation within the IQTIG QA system for surgical site infections had no primary surgical reference.

In addition, case groups with diagnostic codes that assumed no primary association with a surgical surveillance procedure were identified.

The comparison in Table 4 (Tab. 4) shows that the identified cases with the listed diagnostic code groups were significantly more frequently part of the group of cases without a primary surgical reference.

As a result, 96.9% of the cases without a primary surgical reference (n=454, minus subdocumentation=384) were assessed identically. 

In combination with the results in Table 5 (Tab. 5), the correspondingly coded cases were found to have almost no surgical site infections.

In addition, they represented 28.2% of all identically negatively assessed patient cases.

## Discussion and conclusions

With regard to the comparison of both procedures for the surveillance of surgical site infections, the comparison of the IQTIG QA procedure for SSI, which was newly introduced in 2017, with a long-established and proven principle (KISS), provided relevant results.

To avoid the influence of possible first-time errors, the observation period for the comparative study was deliberately set to start from the second year after the introduction of the new IQTIG QA procedure. Extensive training on this procedure had already taken place in 2016 and 2017.

The key message is that the congruence rate of the case assessments has remained constant over the two-year observation period, at 83.7%.

However, the KISS method is only one of several ways to perform adequate surveillance of surgical site infections and has no official national or international status as a gold standard method. Therefore, the concrete interpretation of this congruence rate in relation to the quality or suitability of the IQTIG procedure seems to be difficult and leaves room for subjective discussion.

Further comparisons of numerous parameters between the groups made it possible to identify significantly obvious risk factors for divergent evaluations.

First, it was shown that significantly more of the patient cases assessed as discordant had a primary surgical reference and a positive assessment regarding the occurrence of surgical site infections in general. Both of the identified factors suggest especially those cases were assessed as discordant in which a high probability existed that a surgical site infection was actually present. 

The hypotheses that

1. a poorer health status of patients (group with ASA score = III) or
2. a deviation of the evaluating medical unit from the unit performing the surgery (see Table 2 (Tab. 2)) might have led to increased discordance of assessment, could not be confirmed.

Since the medical personnel primarily responsible for the surveillance work differed in terms of the compared methods [8], [10], the results provide approaches for the basic understanding of surgical site infection surveillance as well as for the recognition of optimization potential.

The summarizing analysis showed some plausible associations within the cases assessed as discordant. In the group of cases that were only positively classified in the QA-SSI procedure, pathogen detection in the blood culture during the time of assessment was significantly more frequent (cf. Table 3 (Tab. 3)). 

Furthermore, there was a clear association with the coding of a pre-existing infection.

In concrete terms, intensified training of the personnel responsible for coding surgical site infections in the context of the surgery and infections elsewhere could increase the precision of recording.

The screening algorithm of the QA-SSI procedure, by which the population was defined, was characterized on real data regarding the composition of the patients preselected in this way. Overall, the fact that 20.3% of all cases (cf. Table 4 (Tab. 4)) did not have a primary surgical association suggests that the strategy of this procedure focuses on sensitivity. The detection of special groups of ICD-10-GM diagnostic codes (Table 5 (Tab. 5)), which were significantly more common in these constellations, would offer the option of modification.

The present research project and its implementation have several limitations. Recording was carried out at a center where intensive training with the new QA-SSI procedure had also been carried out. The follow-up recording analogous to KISS was largely done retrospectively. This is common practice in many instances, but could have led to a loss of information. Blinding of the results during assessment sought to avoid any influences; however, this can never be reliably excluded. The knowledge of the corresponding codes at the time of the post-evaluation by the person in charge has to be considered as a limiting factor. 

In general, the IQTIG QA-SSI surveillance procedure, which was introduced in 2017, presented a consistently high level of agreement in case evaluations measured against a long-established principle, such as KISS. Nevertheless, the methods are not equivalent. 

Possible approaches for optimizing understanding and thus defining of surgical site infections by the medical staff responsible as well as within the system were shown. The end of the test phase of the QA-SSI procedure, which is expected to be in 2021 according to the current status, and possibly an accompanying increased validity through, e.g., extensions of the catalog of measures for participating hospitals, underline the topicality and relevance of the present results. 

The internal documentation rate of the QS-WI procedure as well as the congruence in the comparison of the methods gives exemplary insight into a maximum-care university hospital with many years of quality assurance work and experience. Whether or to what extent these rates vary when hospitals with other levels of care or a nationwide comparison is included cannot be reliably predicted. However, this was not the intention of the analysis, which sought to give a first point-of-reference assessment of the results in comparison, especially against the background of implementation as a control instrument.

In summary, a high degree of congruence between the two recording methods was demonstrated. Furthermore, increased documentation-algorithm efficiency was evident without a loss of documentation of surgical site infections. 

In the group of cases which were evaluated differently, there were plausible associations with systematic sources of error, which could be eliminated at least in part through intensified training.

## Notes

### Competing interests

The authors declare that they have no competing interests.

S. Scheithauer is a member of the expert board of the IQTIG concerning surgical site infections.

### Acknowledgements

Special thanks to the clinical study center of the University Medical Center Goettingen for the methodological and scientific support of the research project and to Dr. Andreas Leha from the Institute for Medical Statistics Goettingen for the statistical data evaluation.

### Funding

The authors did not receive any funds.

### Authorship

S. Rittmeier and R. M. Waeschle share the first authorship.

## Figures and Tables

**Table 1:**
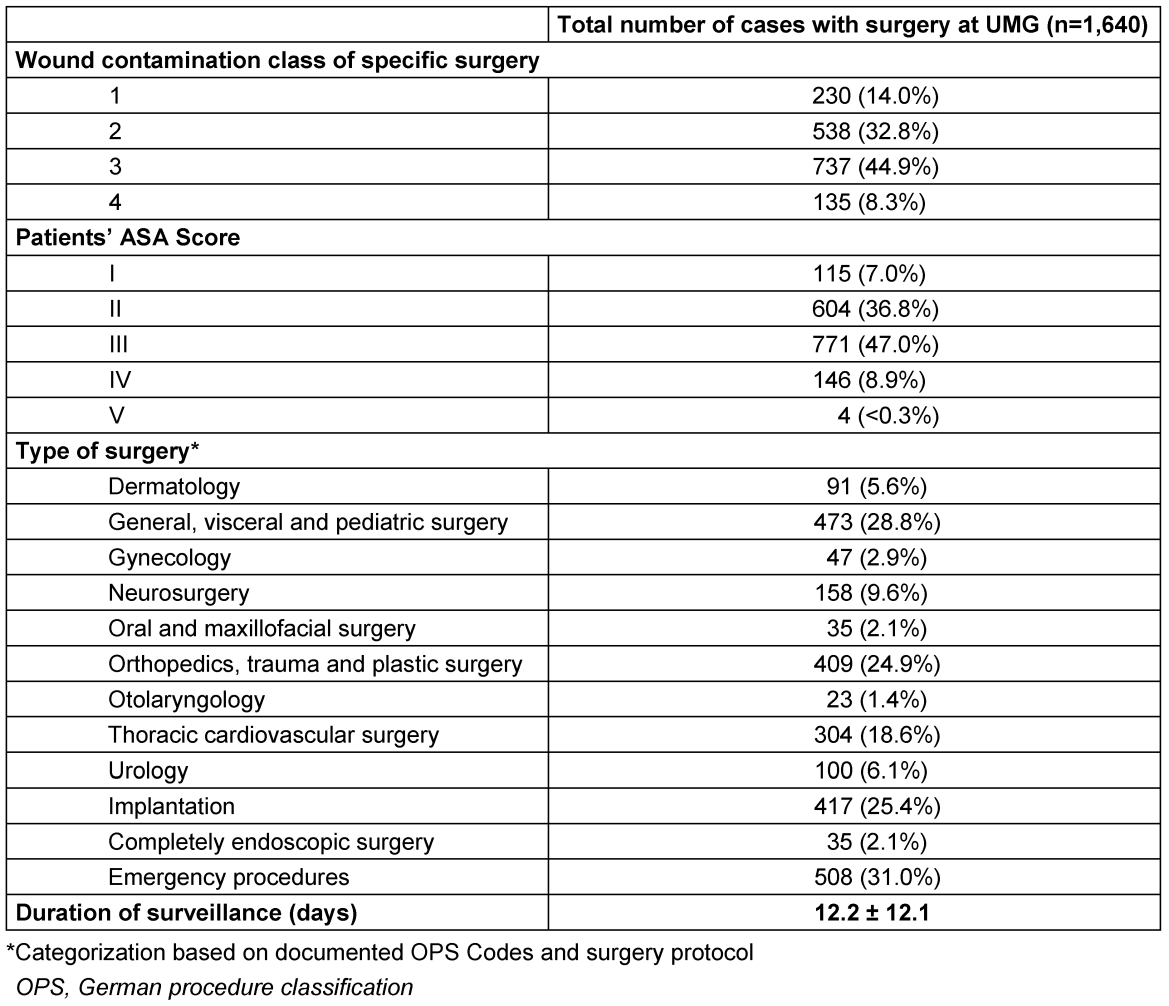
Characterizing overview of the total number of cases with surgery

**Table 2:**
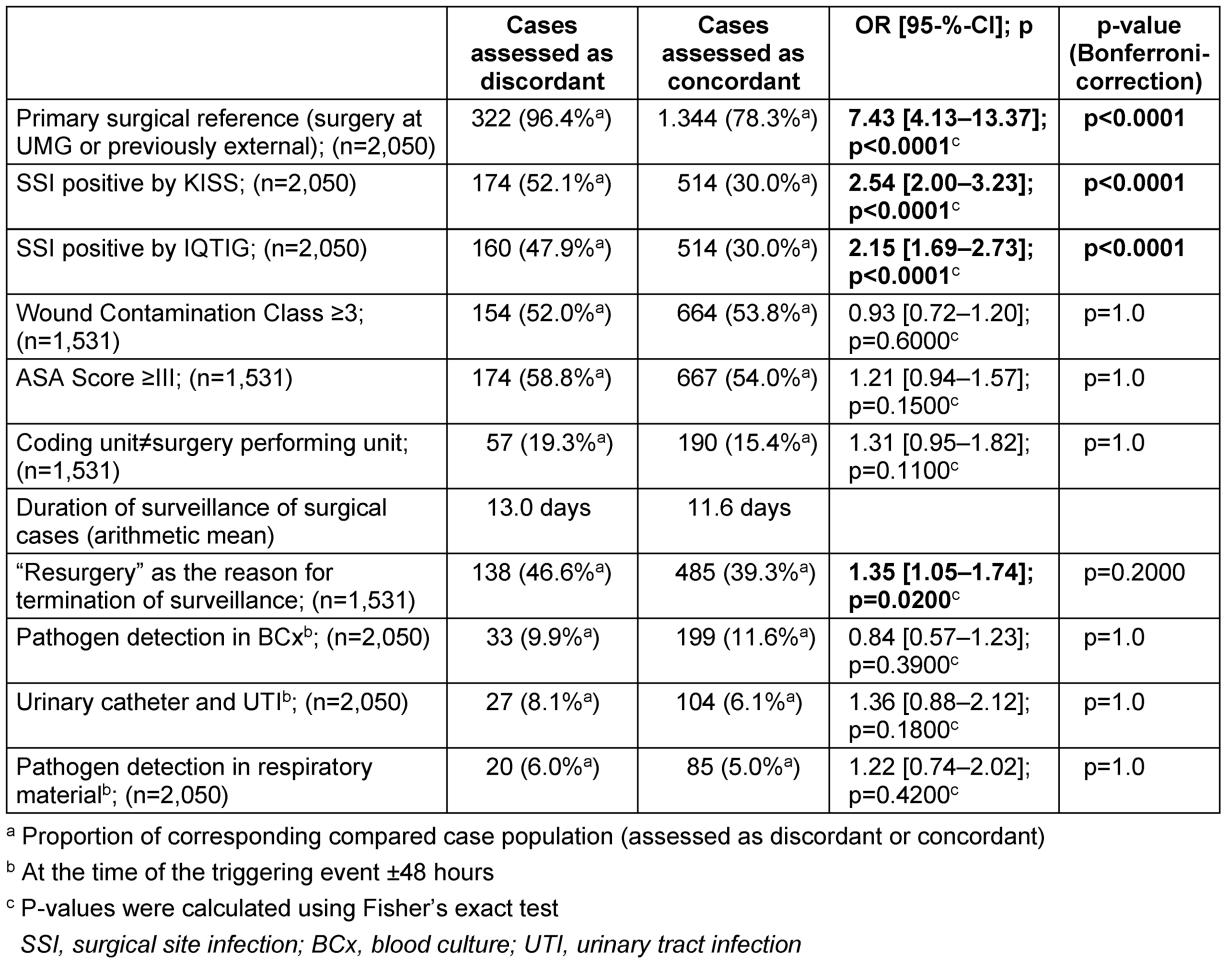
Comparison between cases assessed as discordant and concordant as well as statistical analysis considering different individual characteristics

**Table 3:**
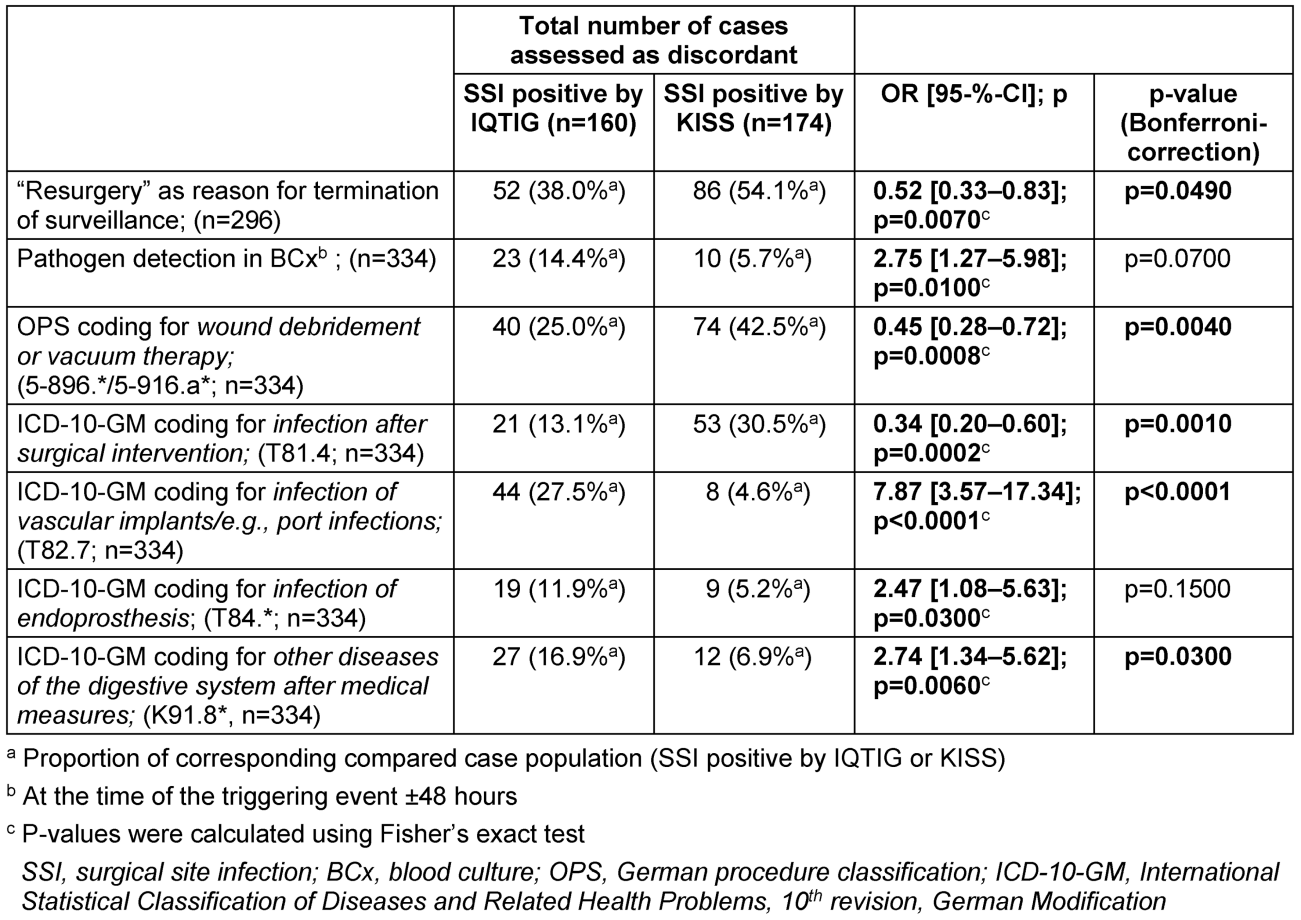
Comparison within the group of cases assessed as discordant as well as statistical analysis considering the different individual characteristics and frequencies of different OPS and ICD-10-GM codes

**Table 4:**
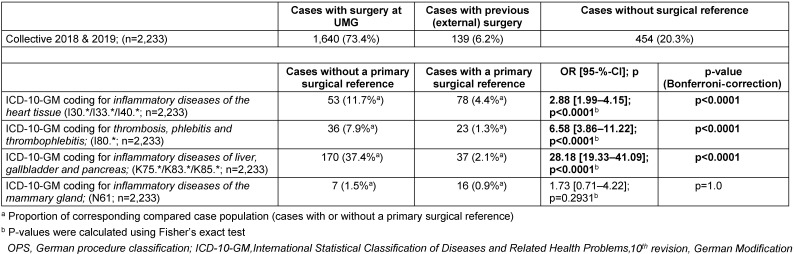
Proportion of cases without a primary surgical reference and intragroup identification of common diagnosis code groups

**Table 5:**
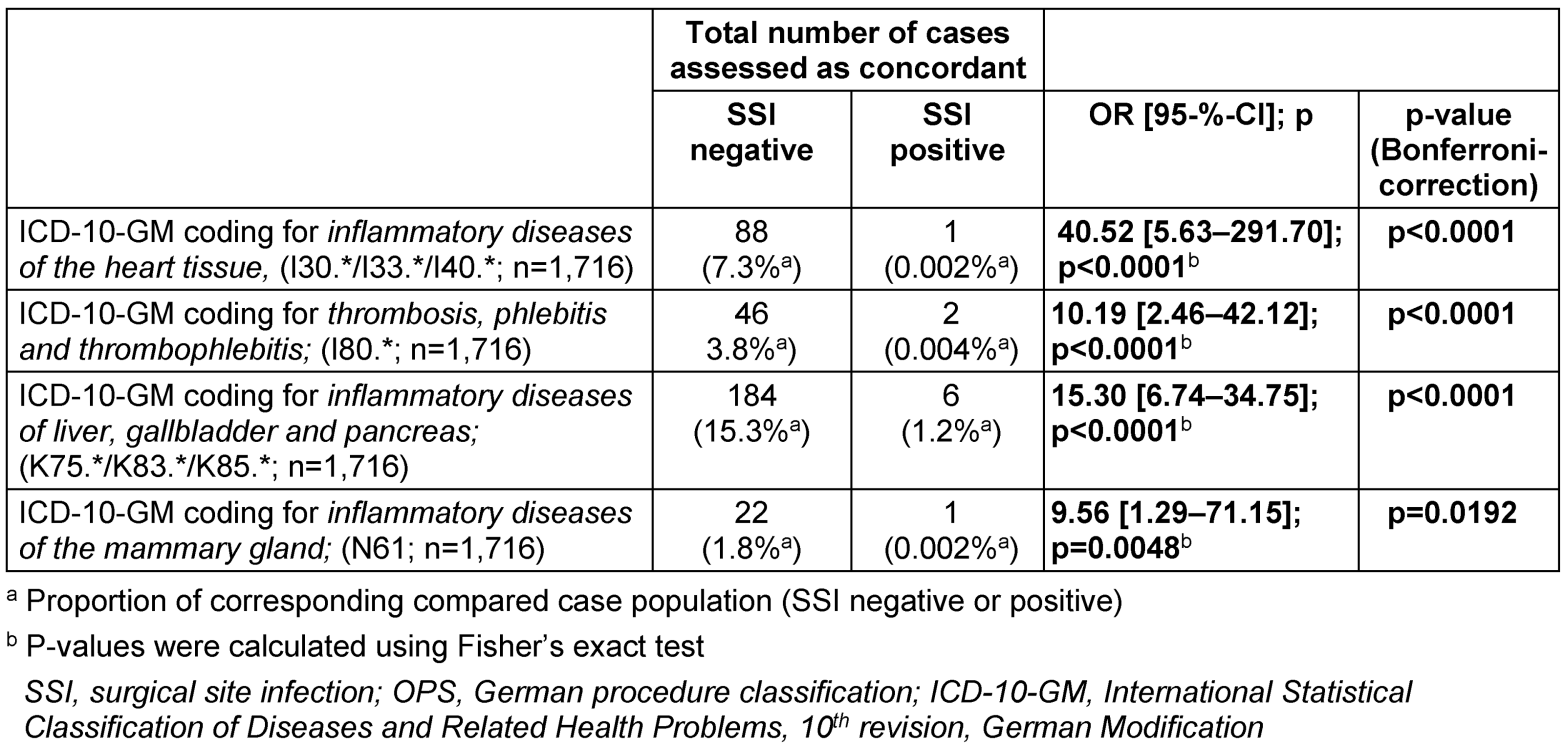
Comparison of case numbers with identified diagnosis code groups within the total concordant assessed cases
